# Supplementary material for: Fatal COVID-19 pulmonary disease involves ferroptosis
Source: Nat Commun. 2024 May 20;15:3816. doi: 10.1038/s41467-024-48055-0 (PMC11106344; doi:10.1038/s41467-024-48055-0)
Supplement: Supplementary file 1 — Supplementary Information [file 41467_2024_48055_MOESM1_ESM.pdf]

Fatal COVID-19 pulmonary disease involves ferroptosis

Baiyu Qiu[1,11], Fereshteh Zandkarimi[1,2,11], Anjali Saqi[3,11], Candace Castagna[4], Hui Tan[1], Miroslav Sekulic[3], Lisa Miorin[5,6], Hanina Hibshoosh[3], Shinya Toyokuni[7,8], Koji Uchida[9], Brent R. Stockwell[1,10*]

[1]Department of Chemistry, Columbia University, New York, NY 10027, USA
[2]Mass Spectrometry Core Facility, Department of Chemistry, Columbia University, New York, NY 10027, USA
[3]Department of Pathology and Cell Biology, Columbia University Irving Medical Center, New York, NY 10032, USA
[4]Institute of Comparative Medicine, Columbia University Irving Medical Center, New York, NY 10032, USA
[5]Department of Microbiology, Icahn School of Medicine at Mount Sinai, New York, NY 10029, USA
[6]Global Health Emerging Pathogens Institute, Icahn School of Medicine at Mount Sinai, New York, NY 10029, USA
[7]Department of Pathology and Biological Responses, Nagoya University Graduate School of Medicine, Nagoya, 466-8550, Japan
[8]Center for Low-temperature Plasma Sciences, Nagoya University, Furo-Cho, Chikusa-ku, Nagoya, 464-8603, Japan
[9]Graduate School of Agricultural and Life Sciences, The University of Tokyo, Tokyo, 113-8657, Japan
[10]Department of Biological Sciences, Columbia University, New York, NY 10027, USA
[11]These authors contributed equally: Baiyu Qiu, Fereshteh Zandkarimi, Anjali Saqi
*Correspondence: bstockwell@columbia.edu (B.R.S.)

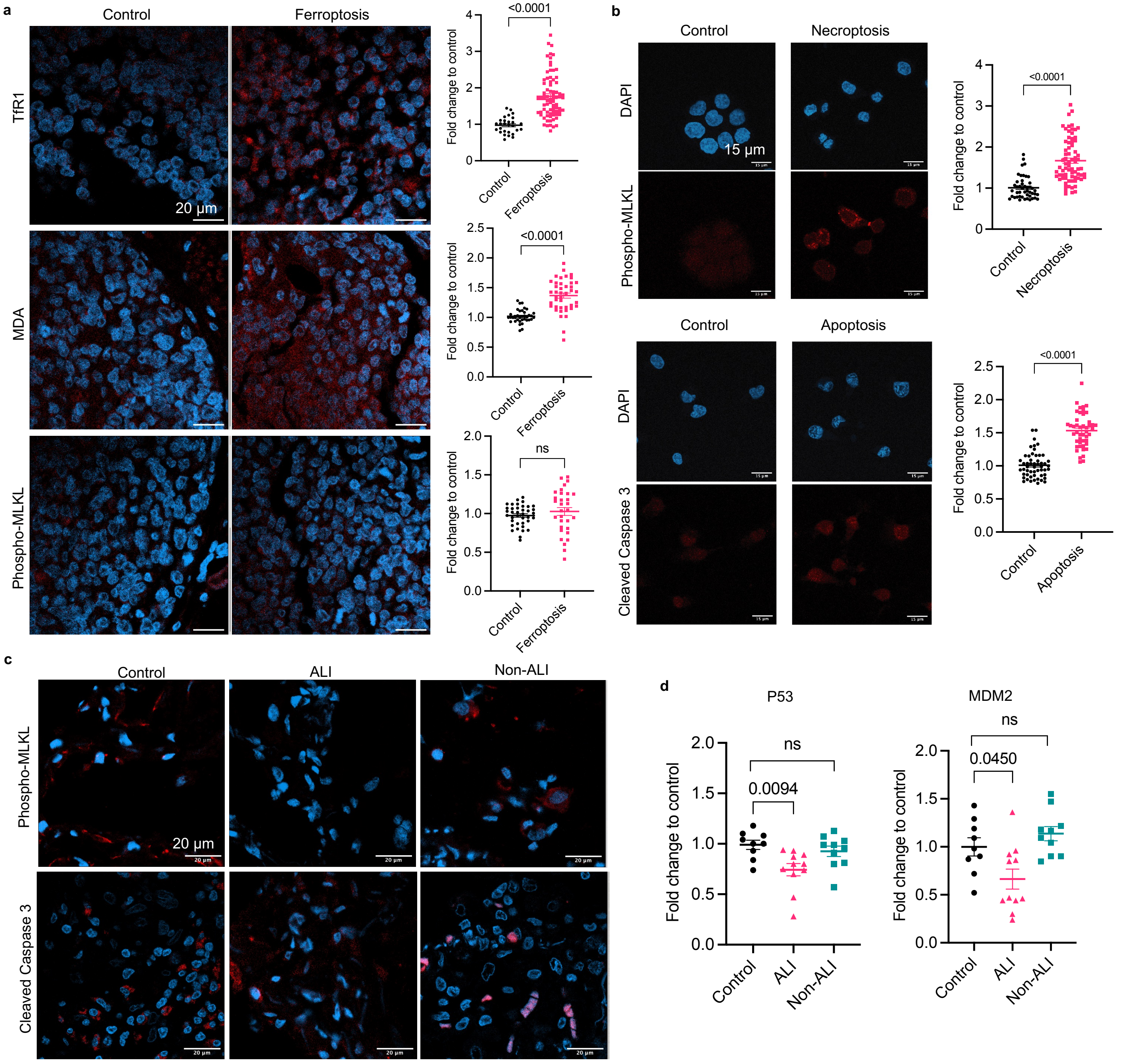

Supplementary Figure 1. Ferroptosis is elevated in post-mortem COVID-19 lungs.

a, Ferroptosis-positive control was analyzed on human cancer xenograft tissue extracted from mice treated with IKE (ferroptosis) or DMSO (control). Representative images of IF staining using anti-TfR1 antibody (clone 3F3-FMA), anti-MDA antibody (clone 1F83), and anti-phospho-MLKL antibody are shown, nucleus in blue and antibody in red. Scale bar = 20 μm. The mean intensity of each antibody is normalized to the control group. Each dot represents a cell. Data shown as mean ± SEM, n = 28-77 for all groups, unpaired two-sided t-test (*p* value indicated). **b,** Necroptosis and apoptosis were induced in HT-29 and HT-1080 cells, respectively. Representative images of IF staining using phospho-MLKL and cleaved Caspase 3 antibodies, nucleus in blue and antibody in red. Scale bar = 20 μm. The mean intensity of each antibody normalized to the control. Each dot represents a cell. Data shown as mean ± SEM, n = 46-72 for all groups, unpaired two-sided t-test (*p* value indicated). **c,** Representative images of phospho-MLKL and cleaved Caspase 3 staining on COVID-19 and control patient samples, nucleus in blue and antibody in red. Scale bar = 20 μm. **d,** Quantification of p53 and MDM2 signals are normalized to the control group. Data shown as mean ± SEM, n = 9 (control), n = 11 (ALI), and n = 10 (non-ALI), one-way ANOVA (*p* value indicated).

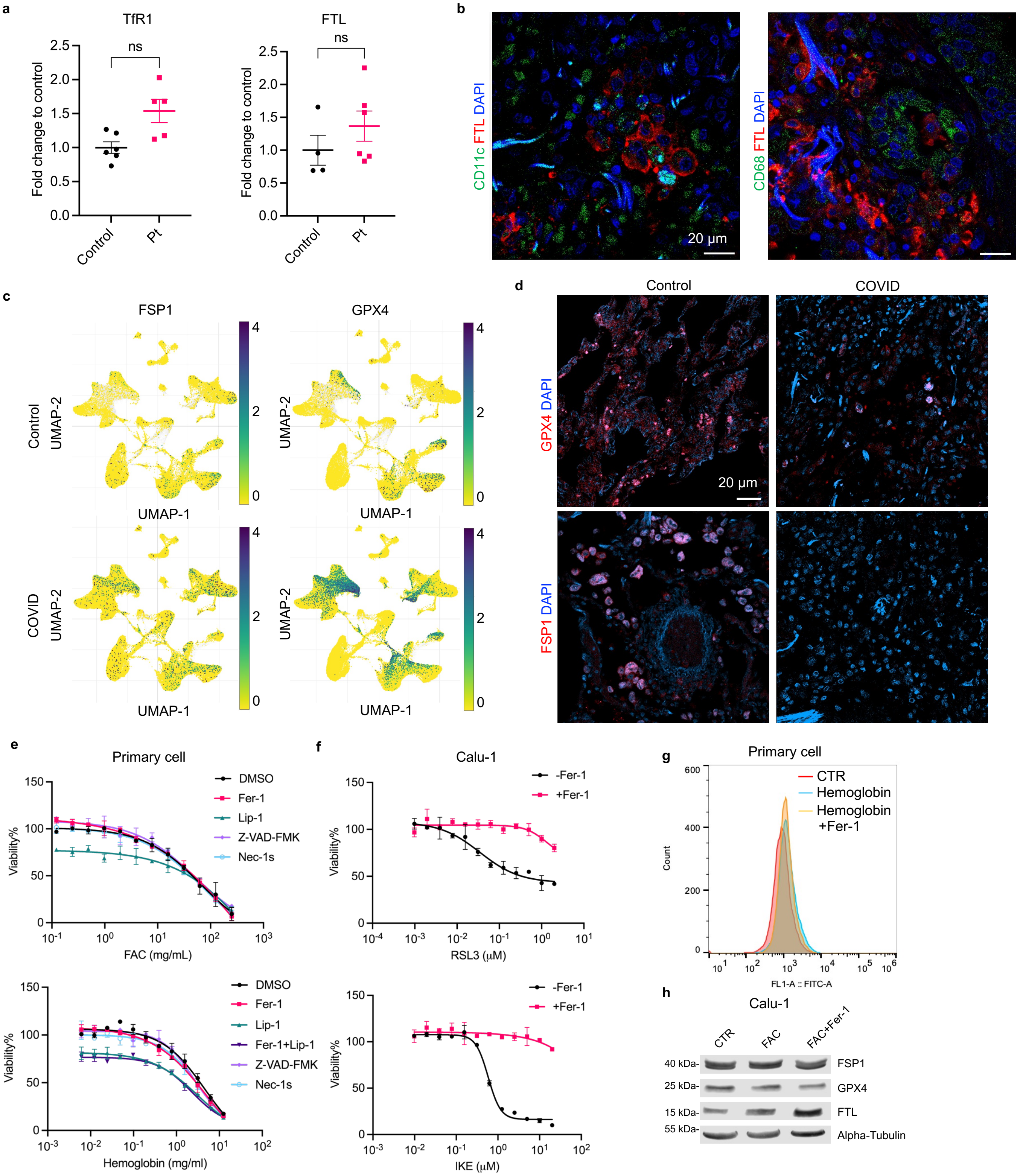
a
TfR1
FTL
ns
Fold change to control
Control
Pt
b
CD11c FTL DAPI
CD68 FTL DAPI
20 μm
c
FSP1
GPX4
Control
COVID
UMAP-1
UMAP-2
d
Control
COVID
GPX4 DAPI
FSP1 DAPI
20 μm
e
Primary cell
DMSO
Fer-1
Lip-1
Z-VAD-FMK
Nec-1s
Fer-1+Lip-1
Viability%
FAC (mg/mL)
Hemoglobin (mg/ml)
f
Calu-1
-Fer-1
+Fer-1
RSL3 (μM)
IKE (μM)
g
Primary cell
CTR
Hemoglobin
Hemoglobin +Fer-1
Count
FL1-A :: FITC-A
h
Calu-1
CTR
FAC
FAC+Fer-1
40 kDa-
25 kDa-
15 kDa-
55 kDa-
FSP1
GPX4
FTL
Alpha-Tubulin

Supplementary Figure 2. Dysregulation of iron homeostasis contributes to ferroptosis.
a, IHC and IF staining of TfR1 (clone H68.4) and FTL in two control groups: healthy lung section from lung neoplasm (control) and healthy lung section from pneumothorax lung (Pt). The mean intensity of TfR1 and FTL signal are normalized to control. Data shown as mean ± SEM, n = 6 (control), n = 5 (Pt) (left panel). n = 4 (control), n = 6 (Pt) (right panel). Unpaired two-sided t-test. **b,** Representative images of severe COVID-19 lung explant stained with anti-FTL antibody and macrophage markers, anti-CD11c and anti-CD68 antibodies. Nucleus in blue, FTL in red, and macrophage marker in green. Scale bar = 20 μm. **c,** Reanalysis of single cell RNA sequencing dataset (GEO, GSE171524). UMAP plot shows the expression of GPX4 and FSP1 across different alveolar cell types in COVID-19 vs. control groups. **d,** Representative images of severe COVID-19 lung explant and control lung stained with anti-GPX4 and anti-FSP1 antibody. Nucleus in blue and antibody in red. Scale bar = 20 μm. **e,** Dose response curve of ferric ammonium citrate (FAC) and hemoglobin in primary lung epithelial cells at 24 h with or without 25 μM of each inhibitor ferrostatin-1 (Fer-1), liproxstatin-1 (Lip-1), Z-VAD-FMK, necrostatin-1s (Nec-1s). Data shown as mean ± SD of n = 2 technical replicates. **f,** Dose response curve of RSL3 and IKE in Calu-1 cells at 24 h with or without 10 μM Fer-1. Data shown as mean ± SD of n = 2 technical replicates. **g,** Lipid peroxidation measure by C11-BODIPY$^{581/591}$ in primary lung epithelial cells treated with 1 mg/mL hemoglobin for 5 h. Data shown as representative result of 2 independent experiments. **h,** Western blot analysis of Calu-1 cells treated with 700 μM FAC with or without 10 μM Lip-1 for 5 h. Whole cell lysate was collected and 40 μg of protein was loaded to each lane. FSP1, GPX4, FTL and α-Tubulin were blotted. Data shown as representative result of 2 independent experiments. Uncropped blots in Supplementary Figure 6.

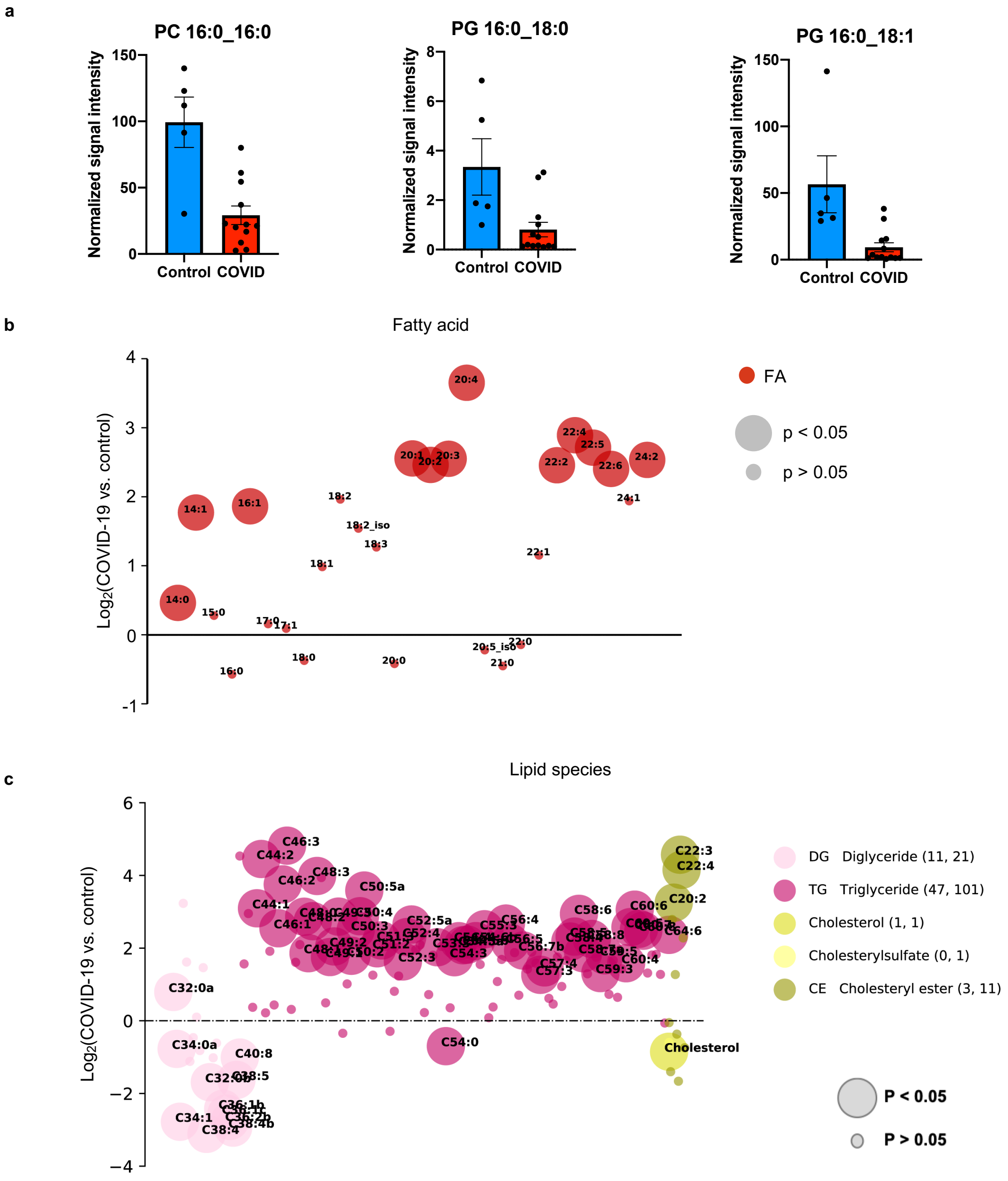

Supplementary Figure 3. Lipidomics reveals lipid dysregulation in fatal COVID-19 lung samples.

a, Bar plots of normalized signal intensities of PC(16:0,16:0) (DPPC), PG(16:0, 18:0), and PG(16:0, 18:1) in control (n = 5) and COVID-19 lung tissues (n = 13). Data shown as mean ± SEM. Bubble plot of $\log_2$ fold changes in abundance of identified **b,** fatty acids and **c,** lipids in COVID-19 lung relative to the control lung. Bubble size represents the FDR-corrected *p* value from the Welch's t-test.

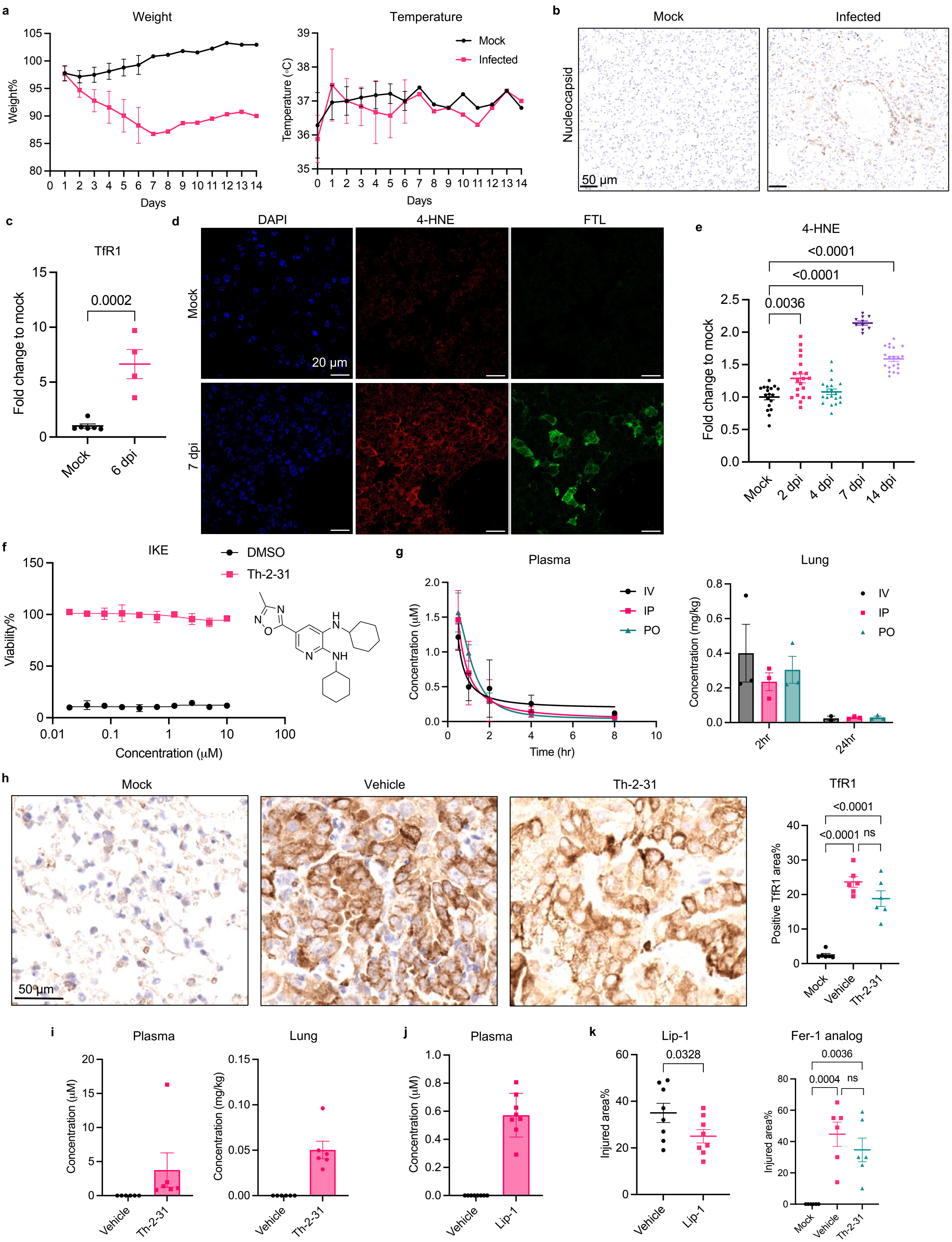
a
Weight
Weight%
Days
Temperature
Temperature (°C)
Mock
Infected
b
Mock
Infected
Nucleocapsid
50 μm
c
TfR1
Fold change to mock
0.0002
Mock
6 dpi
d
DAPI
4-HNE
FTL
Mock
7 dpi
20 μm
e
4-HNE
Fold change to mock
<0.0001
<0.0001
0.0036
Mock
2 dpi
4 dpi
7 dpi
14 dpi
f
IKE
DMSO
Th-2-31
Viability%
Concentration (μM)
g
Plasma
Lung
IV
IP
PO
Concentration (μM)
Time (hr)
Concentration (mg/kg)
2hr
24hr
h
Mock
Vehicle
Th-2-31
50 μm
TfR1
Positive TfR1 area%
<0.0001
<0.0001
ns
i
Plasma
Lung
Concentration (μM)
Concentration (mg/kg)
Vehicle
Th-2-31
j
Plasma
Concentration (μM)
Vehicle
Lip-1
k
Lip-1
Injured area%
0.0328
Fer-1 analog
0.0036
0.0004
ns
Mock

Supplementary Figure 4. Ferroptosis correlates with lung disease severity in a COVID-19 Syrian hamster model.

a, 10-week-old male hamsters were inoculated with 10^5 PFU SARS-CoV-2 and euthanized at several time points to examine lung tissue. Weight and temperature changes post infection are shown as mean ± SD, n = 7, 1-6 dpi; n = 1, 7-14 dpi. **b,** IHC staining of SARS-CoV-2 nucleocapsid protein in mock and infected lungs. Representative images showing nucleus in hematoxylin and nucleocapsid protein in DAB. Scale bar = 50 μm. **c,** TfR1 (clone H68.4) staining on mock and infected lungs. The positive area of TfR1 is normalized to the mock group. Data shown as mean ± SEM, n = 6 (mock), n = 4 (6 dpi), n = 6 (7 dpi), unpaired two-sided t-test (*p* value indicated). **d,** Representative images 4-HNE (clone HNEJ-1) and FTL staining on mock and infected lungs. Scale bar = 20 μm. **e,** The mean intensity of 4-HNE is normalized to the mock section. Each dot represents an image. Data shown as mean ± SEM, n = 10-20 for all sections, one-way ANOVA (*p* value indicated). **f,** HT-1080 cells were co-treated with 3 μM IKE and different doses of ferrostatin-1 analog, Th-2-31 (structure shown) for 24 h. Data shown as mean ± SD of n = 2 technical replicates. **g,** Accumulation of Th-2-31 in hamsters plasma and lungs through 3 different routes of administration: 10 mg/kg for intravenous (IV); 20 mg/kg for intraperitoneal (IP) and oral (PO). **h,** Representative IHC images showing nucleus in hematoxylin and TfR1 (clone H68.4) in DAB in mock, infected-vehicle treated and Th-2-31-treated lungs. Scale bar = 50 μm. Positive area of TfR1 is normalized to the mock group. Data shown as mean ± SEM, n = 6 for each group, one-way ANOVA (*p* value indicated). **i,** The distribution of Th-2-31 in the plasma and lung after treatment with 20 mg/kg Th-2-31 twice daily for 7 days. n = 6 for each group. **j,** The distribution of Lip-1 in the plasma of infected animals treated with 8 mg/kg Lip-1 once daily for 6 days. n = 8 for each group. **k,** Percentage of injured area across each tissue section is shown. Data shown as mean ± SEM, n = 8 (vehicle), n = 8 (Lip-1), unpaired one-sided t-test (p value indicated, left panel)**.** n = 6 (mock), n = 6 (vehicle), n = 6 (Th-2-31), one-way ANOVA (p value indicated, right panel).

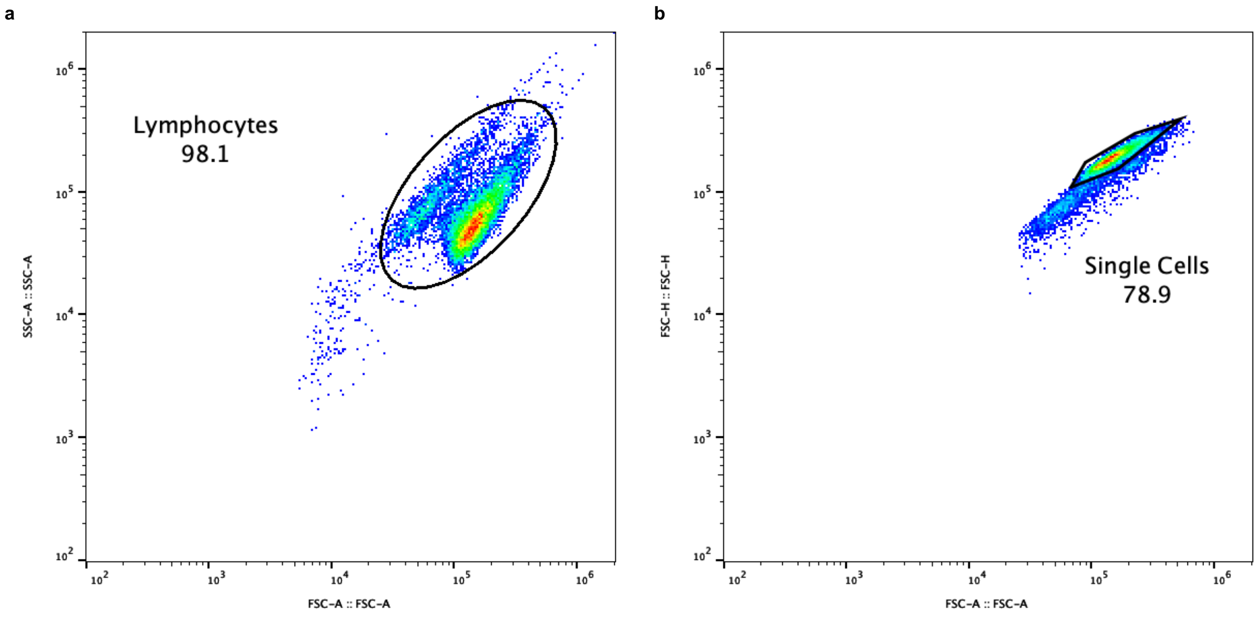

Supplementary Figure 5. Flow cytometry gating method.
a, Live cells (lymphocytes) were gated in SSC-A vs. FSC-A plot and **b,** singlet cells (single cells) were gated in FSC-H vs. FSC-A plot. The same gating method was used for all groups in a single experiment.

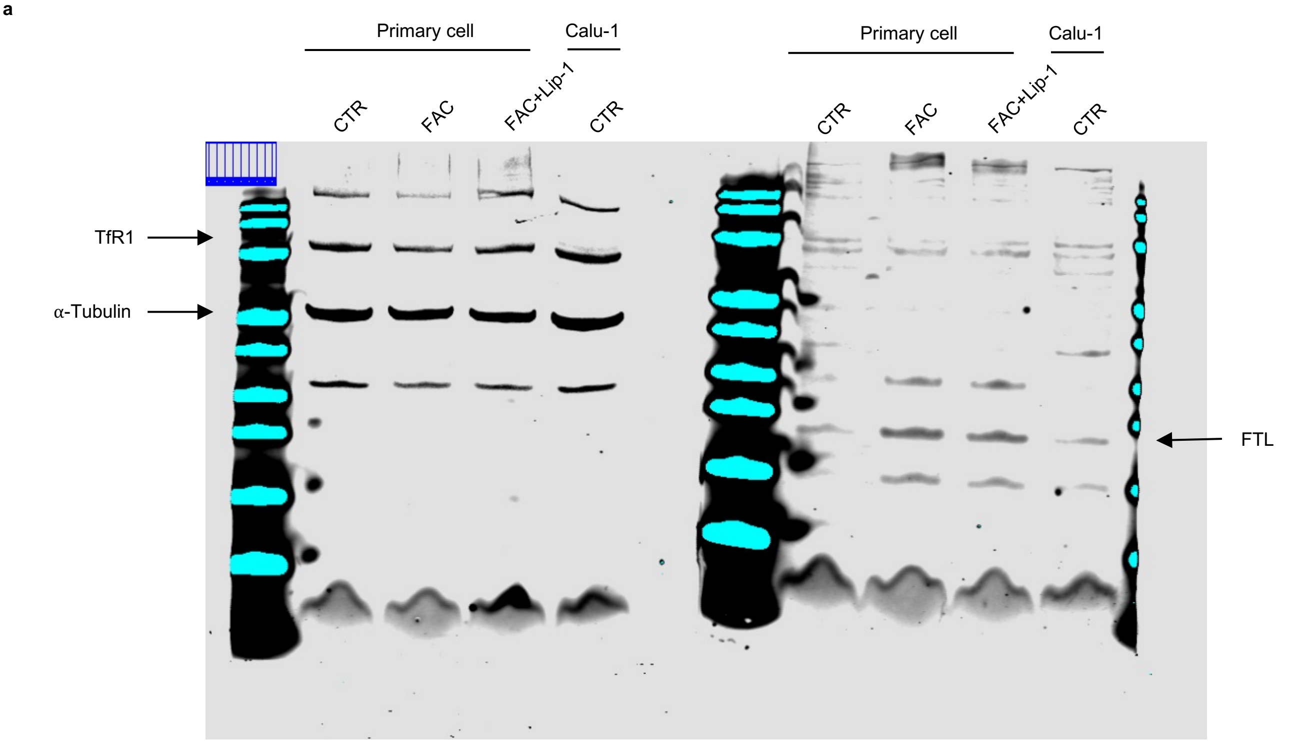

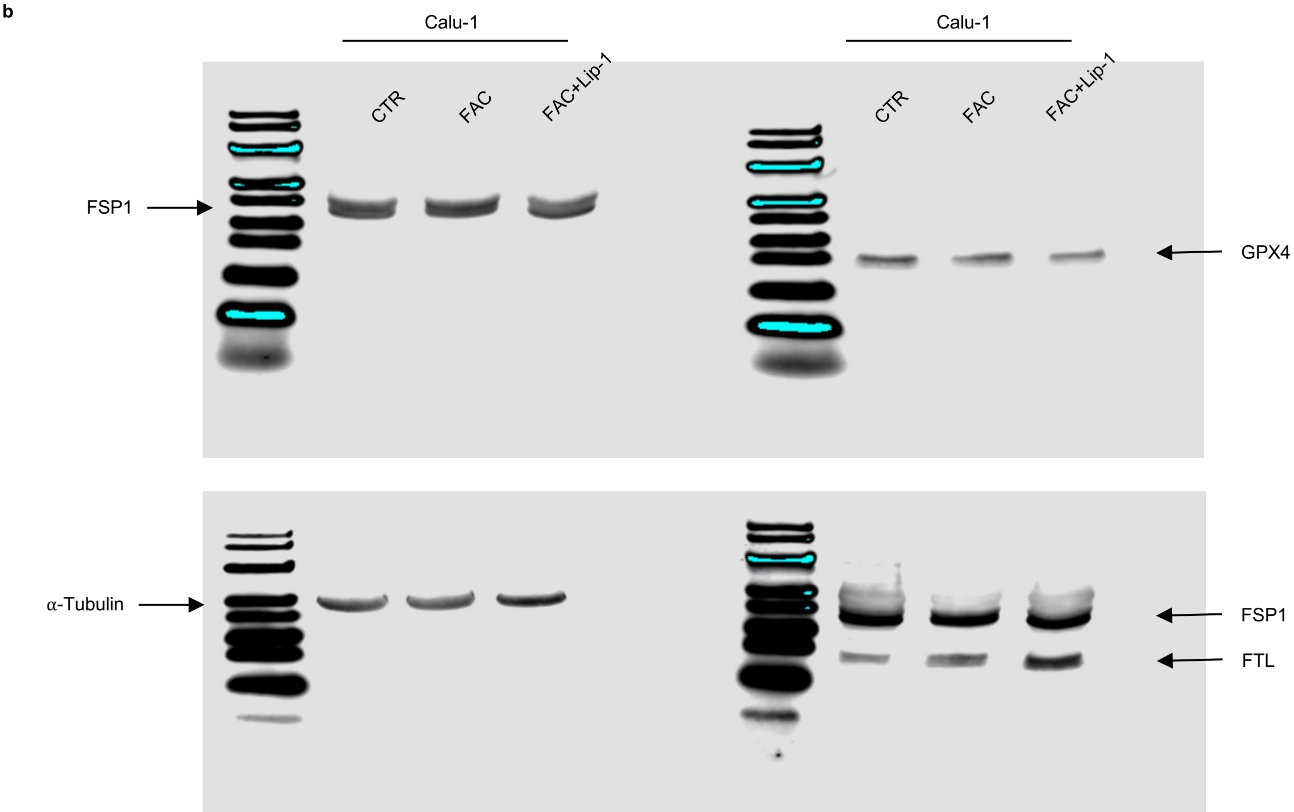

Supplementary Figure 6. Uncropped western blot images.

a, Primary lung epithelial cells were treated with 20 mg/mL FAC with or without 10 μM Lip-1 for 5 h. Whole cell lysate was collected and 40 μg of protein was loaded to each lane. TfR1, FTL, and α-Tubulin were blotted. **b,** Calu-1 cells were treated with 700 μM FAC with or without 10 μM Lip-1 for 5 h. Whole cell lysate was collected and 40 μg of protein was loaded to each lane. FSP1, GPX4, FTL and α-Tubulin were blotted.

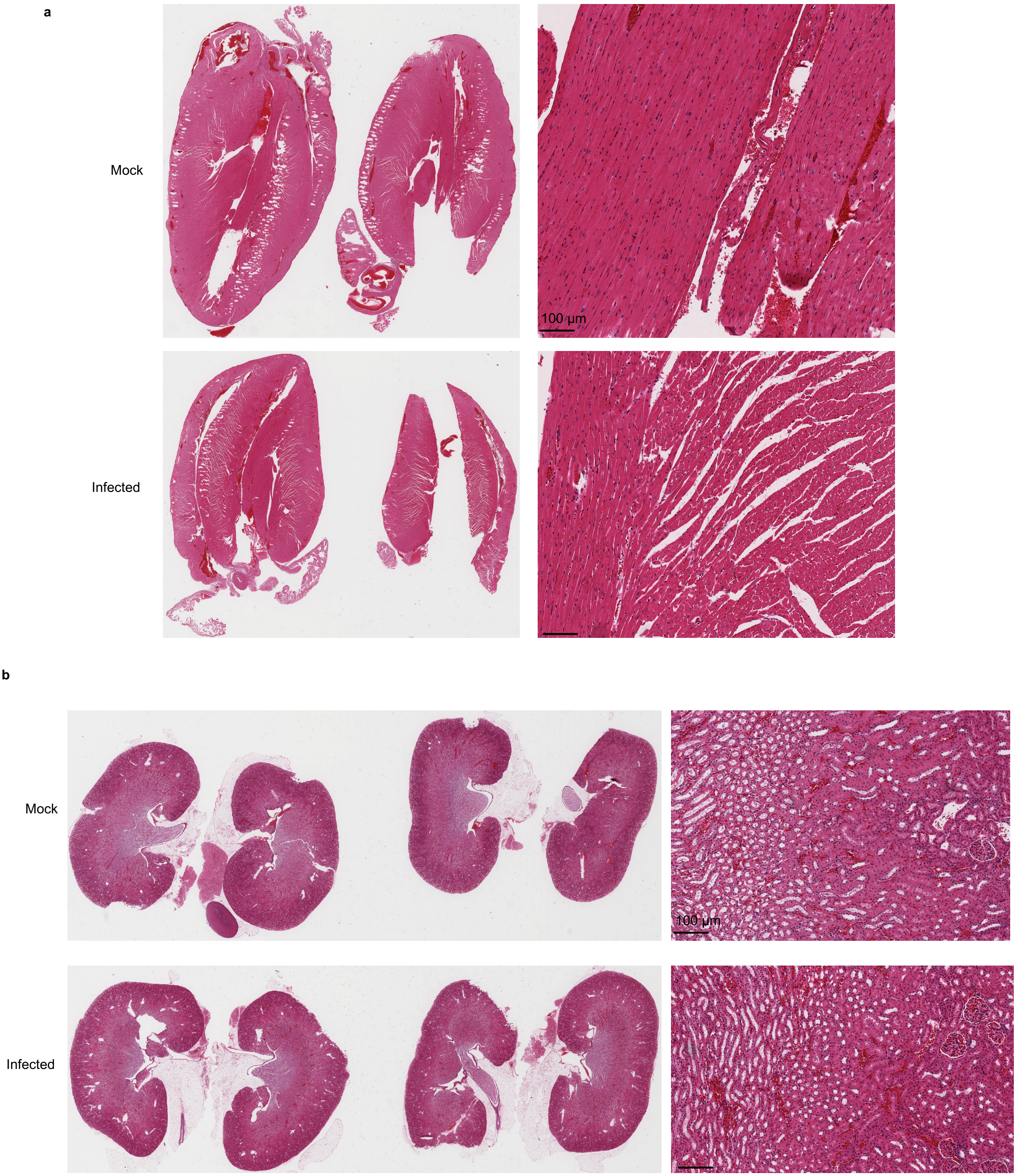

Supplementary Figure 7. H&E images of infected and mock hamster heart and kidney. 10-week-old male hamsters were inoculated with 10^5 PFU SARS-CoV-2 and euthanized at 6 dpi. **a,** The heart and **b,** the kidney were stained with H&E. Scale bar = 100 μm.
