# Supplementary material for: Fatal COVID-19 pulmonary disease involves ferroptosis
Source: Nat Commun. 2024 May 20;15:3816. doi: 10.1038/s41467-024-48055-0 (PMC11106344; doi:10.1038/s41467-024-48055-0)
Supplement: Supplementary file 3 — Description of Additional Supplementary Files [file 41467_2024_48055_MOESM3_ESM.pdf]

Description of Additional Supplementary Files

File Name: Supplementary Data 1
Description: **Sex and gender of human subjects.** The table includes the sex and gender for all human cases involved in this study. The available serum ferritin data are provided.

File Name: Supplementary Data 2
Description: **List of significantly changed lipid species differed between COVID-19 and control groups.** The table includes the name of lipids, ionization mode (positive/negative), retention time, observed m/z values, mass error (Δppm), molecular formula, log2 fold changes (COVID-19/control), and FDR-corrected p values. The isomers are shown as a and b.
