# Supplementary material for: Fatal COVID-19 pulmonary disease involves ferroptosis
Source: Nat Commun. 2024 May 20;15:3816. doi: 10.1038/s41467-024-48055-0 (PMC11106344; doi:10.1038/s41467-024-48055-0)
Supplement: Supplementary file 6 — Reporting Summary [file 41467_2024_48055_MOESM6_ESM.pdf]

nature portfolio

Corresponding author(s): Brent R. Stockwell

Last updated by author(s): 2024/04/01

Reporting Summary

Nature Portfolio wishes to improve the reproducibility of the work that we publish. This form provides structure for consistency and transparency in reporting. For further information on Nature Portfolio policies, see our Editorial Policies and the Editorial Policy Checklist.

Statistics

For all statistical analyses, confirm that the following items are present in the figure legend, table legend, main text, or Methods section.

n/a	Confirmed	
☐	☒	The exact sample size (*n*) for each experimental group/condition, given as a discrete number and unit of measurement
☐	☒	A statement on whether measurements were taken from distinct samples or whether the same sample was measured repeatedly
☐	☒	The statistical test(s) used AND whether they are one- or two-sided<br>*Only common tests should be described solely by name; describe more complex techniques in the Methods section.*
☒	☐	A description of all covariates tested
☒	☐	A description of any assumptions or corrections, such as tests of normality and adjustment for multiple comparisons
☐	☒	A full description of the statistical parameters including central tendency (e.g. means) or other basic estimates (e.g. regression coefficient) AND variation (e.g. standard deviation) or associated estimates of uncertainty (e.g. confidence intervals)
☐	☒	For null hypothesis testing, the test statistic (e.g. *F*, *t*, *r*) with confidence intervals, effect sizes, degrees of freedom and *P* value noted<br>*Give P values as exact values whenever suitable.*
☒	☐	For Bayesian analysis, information on the choice of priors and Markov chain Monte Carlo settings
☒	☐	For hierarchical and complex designs, identification of the appropriate level for tests and full reporting of outcomes
☒	☐	Estimates of effect sizes (e.g. Cohen's *d*, Pearson's *r*), indicating how they were calculated

Our web collection on statistics for biologists contains articles on many of the points above.

Software and code

Policy information about availability of computer code

Data collection	Immunofluorescence images were obtained using Zeiss Zen Blue v2.1. IHC images were obtained using Leica AT2. Flow cytometry data was collected using Beckman CytoFLEX System B4-R0-V0 flow cytometer.
Data analysis	Image analysis was performed using CellProfiler (v4.0.7, RRID:SCR_007358) and QuPath (v0.4.3, RRID:SCR_018257). The raw LC-MS data files were analyzed using MassLynx software (v4.2, Waters), the XCMS package in the R (v4.0) environment and MetaboAnalyst (v3.0). Statistical analysis was performed using GraphPad Prism 9. Flow cytometry data were analyzed using FlowJo (v10.8).

For manuscripts utilizing custom algorithms or software that are central to the research but not yet described in published literature, software must be made available to editors and reviewers. We strongly encourage code deposition in a community repository (e.g. GitHub). See the Nature Portfolio guidelines for submitting code & software for further information.

Data

Policy information about availability of data

All manuscripts must include a data availability statement. This statement should provide the following information, where applicable:

- Accession codes, unique identifiers, or web links for publicly available datasets
- A description of any restrictions on data availability
- For clinical datasets or third party data, please ensure that the statement adheres to our policy

The mass spectrometry data generated in this study have been deposited in the EMBL-EBI MetaboLights database under accession code MTBLS3107. The rest data

generated in this study are provided in the Supplementary Information/Source Data file. The single cell RNA-sequencing data used in this study are available in the GEO database under accession code GSE171524 [https://doi.org/10.1038/s41586-021-03569-1].

Research involving human participants, their data, or biological material

Policy information about studies with human participants or human data. See also policy information about sex, gender (identity/presentation), and sexual orientation and race, ethnicity and racism.

Reporting on sex and gender	Both male and female were equally involved. No sex- and gender-based analyses have been performed because the sex and gender does not influence the results. All subjects involved in this study are fatal COVID-19 cases and were selected based on pulmonary pathological features only.
Reporting on race, ethnicity, or other socially relevant groupings	This study didn't use race, ethnicity, or other socially relevant groupings.
Population characteristics	Severe COVID-19 lung autopsies were obtained from Columbia University tissue bank and collected from patients deceased from respiratory failure caused by SARS-CoV-2 infection. Mild COVID-19 lung biopsies were selected from lung transplants. Control lungs were selected from resections of pneumothorax lungs and neoplastic lungs that were not infected with SARS-CoV-2. Non-COVID-19 lung autopsies were collected from patients deceased from respiratory failure before the pandemic. COVID and control groups are age-matched and the detailed age range were provided in Supplementary Data 1.
Recruitment	Patient autopsy samples were examined for pathological features and recruited based on the types of lung injury present.
Ethics oversight	The study involving human subject was conducted under the Institutional Review Board of Columbia University (AAAT0388, approved 04/28/2020).

Note that full information on the approval of the study protocol must also be provided in the manuscript.

Field-specific reporting

Please select the one below that is the best fit for your research. If you are not sure, read the appropriate sections before making your selection.

☒ Life sciences ☐ Behavioural & social sciences ☐ Ecological, evolutionary & environmental sciences

For a reference copy of the document with all sections, see nature.com/documents/nr-reporting-summary-flat.pdf

Life sciences study design

All studies must disclose on these points even when the disclosure is negative.

Sample size	Sample size was calculated using G Power of 80-90%, p-value of 0.05, and effect size based on the staining data from pilot study to ensure statistical significance.
Data exclusions	No data were excluded from the study.
Replication	All experiments were repeated at least 2 or 3 times as described in figure legends. All replication attempts were repeatable and reproducible with consistent observations.
Randomization	All animals were randomized into different treatment groups. During lipidomics analysis, samples were randomized for the order of running. Tissue samples were randomized during processing, staining, and image acquisition. Cell experiments were not relevant to randomization because cells in vitro are inherentely randomized.
Blinding	Investigators were blinded to group allocation during data collection and analysis.

Reporting for specific materials, systems and methods

We require information from authors about some types of materials, experimental systems and methods used in many studies. Here, indicate whether each material, system or method listed is relevant to your study. If you are not sure if a list item applies to your research, read the appropriate section before selecting a response.

Materials & experimental systems

n/a	Involved in the study
☐	☒ Antibodies
☐	☒ Eukaryotic cell lines
☒	☐ Palaeontology and archaeology
☐	☒ Animals and other organisms
☒	☐ Clinical data
☒	☐ Dual use research of concern
☒	☐ Plants

Methods

n/a	Involved in the study
☒	☐ ChIP-seq
☐	☒ Flow cytometry
☒	☐ MRI-based neuroimaging

Antibodies

Antibodies used	TfR1 antibody (clone 3F3-FMA), originated from the Stockwell lab18, validated in this study using human DLBCL xenograft tissue; MDA adduct antibody (clone 1F83), originated from the Uchida lab and validated in this study using human DLBCL xenograft tissue19; phospho-MLKL antibody (phospho S358, clone EPR9514), Abcam ab187091, RRID:AB_2619685; cleaved Caspase 3 antibody (Asp175), Cell Signaling Technology 9661L, RRID:AB_2341188; TfR1 antibody (clone H68.4), Santa Cruz sc-65882, RRID:AB_1120670; cleaved Gasdermin D antibody (clone E7H9G), Cell Signaling Technology 36425, RRID:AB_2799099; 4-HNE antibody (clone HNEJ-1), originated from the Toyokuni lab36; p53 antibody (clone D0-1), Santa Cruz sc-126, RRID:AB_628082; MDM2 antibody (clone D1V2Z), Cell Signaling Technology 86934, RRID:AB_2784534; FTL antibody, Proteintech 10727-1-AP, RRID: AB_2278673; CD11c antibody (clone N418) conjugated with Alexa Fluor 488, Thermo Fisher Scientific 53-0114-82, RRID:AB_469903; CD68 antibody (clone FA-11), Abcam ab53444, RRID:AB_869007; [illegible]-Tubulin antibody (clone DM1A), Santa Cruz sc-32293, RRID: AB_628412; FSP1 antibody, Proteintech 20886-1-AP, RRID:AB_2878756; GPX4 antibody, Abcam ab125066, RRID:AB_10973901; goat anti-Mouse IgG (H+L) antibody Alexa Fluor 594, Thermo Fisher Scientific A-11032, RRID: AB_2534091; goat anti-Rabbit IgG (H+L) antibody Alexa Fluor 594, Thermo Fisher Scientific A-32740, RRID:AB_2762824; goat anti-Rat IgG (H+L) antibody Alexa Fluor 488, Cell Signaling Technology 4416, RRID:AB_10693769.
Validation	All antibodies originated from research labs were previously validated in published studies and in this study using human DLBCL xenograft tissue or cell line. All commercially available antibodies were validated by manufacturers. Research Resource Identifiers (RRID) confirming validation and linking to clone, clonality and species from manufacturers' websites are provided above.

Eukaryotic cell lines

Policy information about cell lines and Sex and Gender in Research

Cell line source(s)	HT-1080, HT-29, and Calu-1 cell lines were obtained from Columbia University Cancer Cell Bank. Human primary lung epithelial cell was obtained from ATCC (PCS300010).
Authentication	All cell lines were authenticated through STR profiling by the Cell Bank or ATCC.
Mycoplasma contamination	All cell lines were tested negative of mycoplasma contamination.
Commonly misidentified lines (See ICLAC register)	None.

Animals and other research organisms

Policy information about studies involving animals; ARRIVE guidelines recommended for reporting animal research, and Sex and Gender in Research

Laboratory animals	10-week-old male Golden Syrian hamsters (Mesocricetus auratus; outbred hamster strain 049, Charles River Labs).
Wild animals	None.
Reporting on sex	Findings apply to only one sex. Only male animals were used because this study did not investigate the sex difference in pulmonary disease induced by SARS-CoV-2 infection.
Field-collected samples	None.
Ethics oversight	This study was approved by the Institutional Animal Care and Use Committee of Columbia University (AC-AABT6656, approved 12/15/2022).

Note that full information on the approval of the study protocol must also be provided in the manuscript.

Flow Cytometry

Plots

Confirm that:

☒ The axis labels state the marker and fluorochrome used (e.g. CD4-FITC).

☒ The axis scales are clearly visible. Include numbers along axes only for bottom left plot of group (a 'group' is an analysis of identical markers).

☒ All plots are contour plots with outliers or pseudocolor plots.

☒ A numerical value for number of cells or percentage (with statistics) is provided.

Methodology

Sample preparation	Cells were seeded in 6-well plates at 0.5 million cells per well and incubated overnight. Cells were treated with conditions specified in figure legends and washed once with HBSS. Cells were then incubated with HBSS containing 2 µM C11-BODIPY581/591 at 37 ºC for 30 min in dark. Cells were trypsonized, washed once with HBSS, and resuspended in fresh HBSS for flow cytometry analysis.
Instrument	Beckman CytoFLEX System B4-R0-V0 flow cytometer
Software	FlowJo v10.8
Cell population abundance	A single cell line was used for each experiment so no cell sorting was used.
Gating strategy	Cells were gated for live cells in SSC-A vs FSC-A plot and then for singlet cells in FSC-H vs FSC-A plot. The FITC-A histogram of singlet cells was plotted for comparison between treatment group and control group.

☒ Tick this box to confirm that a figure exemplifying the gating strategy is provided in the Supplementary Information.
